# Oral Corticosteroid Use and Its Associated Complications in Patients With Sarcoidosis: A Nationwide Claims Study From Japan

**DOI:** 10.7759/cureus.93054

**Published:** 2025-09-23

**Authors:** Koichi Miyashita, Keita Hashimoto, Shotaro Maeda, Takafumi Suda

**Affiliations:** 1 Internal Medicine, Hamamatsu University School of Medicine, Hamamatsu, JPN; 2 Medical Affairs, Kyorin Pharmaceutical Co. Ltd., Tokyo, JPN

**Keywords:** claims data, complications, corticosteroid, japan, sarcoidosis

## Abstract

Background: Corticosteroids are the first-line therapy for sarcoidosis due to their potent anti-inflammatory effects, providing symptomatic relief and slowing disease progression. In Japan, the association between cumulative corticosteroid exposure and the risk of steroid-related complications in patients with sarcoidosis remains unclear. In this study, we aimed to investigate real-world oral corticosteroid (OCS) dosing patterns and their association with adverse outcomes among patients with sarcoidosis using a nationwide claims database.

Methods: This retrospective cohort study used the Deidentified Scrubbed Claims Healthcare Database in Japan, covering approximately 20 million individuals between April 2014 and October 2023. Patients aged ≥18 years with a diagnosis of sarcoidosis were included. After applying eligibility criteria, patients who initiated OCS were matched 1:3 by index month with those who did not. Patients were followed for up to three years. Steroid-related complications were identified using diagnosis and treatment codes. The cumulative incidence of steroid-related complications was estimated using the Kaplan-Meier method, and multivariable Cox proportional hazards models were used to assess associations between three-year cumulative OCS dose and outcomes.

Results: Among 25,779 adults with sarcoidosis, 585 patients who initiated OCS were matched 1:3 with 1696 non-OCS patients, resulting in 2281 patients included in the analysis. Among OCS users, 401 (68.5%) initiated treatment within six months of diagnosis, with 220 (37.6%) receiving 30 mg/day as the most common initial dose. Compared with the non-OCS group, the hazard ratio (HR) for vertebral fracture was 1.43 (95% confidence interval (CI): 0.74-2.77) in the 1-4999 mg group and 2.13 (95% CI: 1.01-4.48) in the ≥5000 mg group. For pneumonia, HRs were 5.87 (95% CI: 4.43-7.79) and 13.47 (95% CI: 10.01-18.12), respectively. Cumulative OCS exposure in both the 1-4999 mg and ≥5000 mg groups was significantly associated with increased risks of herpes zoster, insomnia, hypertension, hyperlipidemia, and type 2 diabetes mellitus. Glaucoma was significantly associated only in the 1-4999 mg group, while cataract showed no significant association in either group.

Conclusion: Using a large-scale Japanese claims database, to the best of our knowledge, this study is the first to demonstrate that OCS use in sarcoidosis is associated with increased risks of dose-dependent complications. These findings emphasize the need for individualized treatment strategies and highlight the importance of expanding therapeutic options beyond corticosteroids.

## Introduction

Sarcoidosis is an inflammatory disease of unknown etiology, characterized by non-caseating granulomas, most commonly affecting the lungs and lymph nodes [1]. Clinical manifestations vary depending on the organs involved. Pulmonary involvement typically presents with respiratory symptoms such as cough and dyspnea; however, some patients may exhibit systemic, non-organ-specific symptoms, including fever and fatigue [2]. Spontaneous remission can occur; however, sarcoidosis may progress to a chronic or refractory form, significantly impairing patients’ quality of life (QOL).

Pharmacological treatment - primarily corticosteroids and immunosuppressive agents - remains the standard approach for sarcoidosis management [3]. In Japan, corticosteroids are currently the only medication approved under the national health insurance system. Due to their potent anti-inflammatory effects, corticosteroids serve as first-line therapy, relieving acute symptoms and slowing disease progression [3]. However, adverse effects associated with prolonged use pose major clinical concerns.

Patients with autoimmune diseases and asthma who receive high-dose corticosteroids face elevated risks of impaired glucose metabolism, bone disorders, and infections [4-7]. A United Kingdom (UK) study reported that vertebral fracture rates were 2.6 times higher in oral corticosteroid (OCS) users than in non-users, with the risk increasing in a dose-dependent manner [8]. Data from the United States (US) support these findings, linking corticosteroid use to an increased risk of fractures in patients with inflammatory diseases, including sarcoidosis [9]. Swedish registry analyses also demonstrated a higher infection risk associated with corticosteroid therapy in sarcoidosis, which adversely affected QOL and long-term outcomes [10].

Understanding real-world corticosteroid prescribing patterns is essential for safe and effective clinical practice. International studies in newly diagnosed sarcoidosis have reported that 41% of patients required treatment within two years, with half initiating prednisone within 30 days; prescribed doses varied based on baseline characteristics [11]. In South Korea, 78% of patients received systemic corticosteroids within one year, and 50.9% continued treatment for more than 30 days. The mean daily dose during the initial 30 days was 33.0 ± 25.7 mg in prednisone-equivalent terms [12]. These findings highlight the need to understand corticosteroid use in sarcoidosis. However, large-scale epidemiological data from Japan remain limited. Furthermore, the association between cumulative corticosteroid dose and steroid-related complications in patients with sarcoidosis has not been clearly established.

Therefore, in this study, we aimed to evaluate actual cumulative corticosteroid doses and their association with steroid-related complications in newly diagnosed patients with sarcoidosis in Japan, using a large-scale nationwide database. The findings are intended to inform risk assessment and support improved treatment strategies in clinical practice.

## Materials and methods

Data source and study design

This retrospective cohort study used a Japanese administrative claims database provided by Deidentified Scrubbed (DeSC) Healthcare Inc., a commercial database provider, to evaluate cumulative OCS exposure and its association with OCS-related complications in patients newly diagnosed with sarcoidosis. The database contains data from three major national health insurance systems in Japan - the National Health Insurance, Health Insurance Societies, and the Late Elderly Health Insurance System - covering approximately 20 million individuals between April 2014 and October 2023.

**Figure 1:**
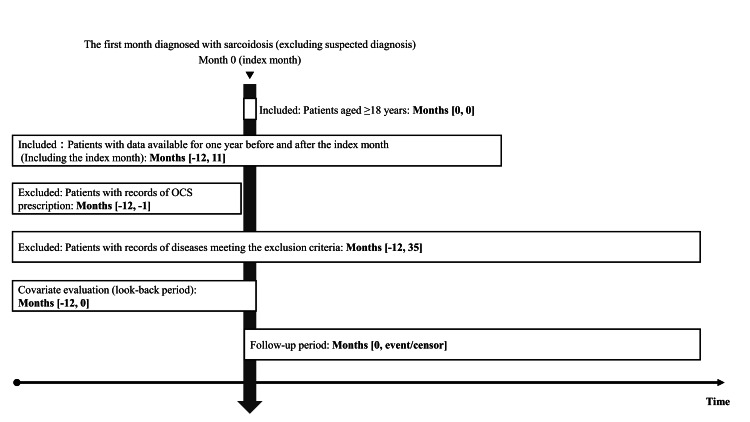
Overall study design Schematic representation of the patient selection process and study timeline. The index month (Month 0) was defined as the first recorded diagnosis of sarcoidosis (excluding suspected cases). Eligible patients were ≥18 years of age with continuous enrollment for ≥12 months before and after the index month. Patients were excluded if they had OCS prescriptions during the 12 months prior to the index month or any diagnosis meeting predefined exclusion criteria from 12 months before to 35 months after the index month. Baseline covariates were assessed during the 12-month pre-index period, and patients were followed from the index month until the occurrence of an outcome event or censoring. OCS: oral corticosteroid This figure was created by the authors using data from the DeSC Healthcare claims database. No permission required.

Patients aged 18 years or older with a diagnosis of sarcoidosis, defined by the International Classification of Diseases, 10th Revision (ICD-10) code D86, were included. The month of the first recorded sarcoidosis diagnosis was defined as the index month. Eligible patients were required to have at least one year of continuous data both before and after the index month, no prior history of OCS prescriptions, and no predefined exclusionary conditions (Figure 1 and Table 1). For patients who received OCS prescriptions, the index month was redefined as the month in which OCS was first prescribed. To address immortal time bias in the OCS group - arising from not accounting for events occurring between OCS prescription and the original index month - patients who received OCS were matched 1:3 with those who did not, using the original index month. Subsequently, patients who met the eligibility criteria were followed up for three years from the index month.

**Table 1:** Codes used to define the exclusion criteria The International Classification of Diseases, 10th Revision (ICD-10) codes were used to define the exclusion criteria. Diagnoses were confirmed based on the presence of standardized disease names in the claims database (Shoubyoumei Kihon Meishou). Segmental vitiligo was specifically identified based on disease name entries.

Exclusion condition	ICD-10 code
Respiratory cancer	C3x
Multiple sclerosis	G35
Rheumatoid arthritis	M05-M06, 08
Ankylosing spondylitis	M45
Systemic lupus erythematosus	M30-36
Psoriasis	L40
Crohn’s disease	K50
Ulcerative colitis	K51
Sjogren’s syndrome	M30-36
Systemic scleroderma	M30-36
Dermatomyositis	M30-36
Polymyositis	M30-36
Thromboangiitis obliterans	I731
Behçet’s disease	M30-36
Pemphigus	L00, L10x, L121, L129, L133, Q828
Vitiligo	L80 (excluding age-related vitiligo; cases extracted only if disease name includes segmental vitiligo)

Study variables

Patient characteristics included age, sex, Charlson Comorbidity Index (CCI) [13], length of the follow-up period after the index month, presence of OCS-related outcomes during the 12 months prior to the index month, and cumulative OCS dose during the post-index follow-up period.

OCS-related outcomes were defined based on previous studies [14,15] and our prior epidemiological research [16]. These outcomes included vertebral fracture, pneumonia, herpes zoster, urinary tract infection, hypertension, hyperlipidemia, type 2 diabetes mellitus, insomnia, glaucoma, and cataract. The inclusion of outcomes such as type 2 diabetes mellitus, insomnia, and herpes zoster was further supported by our earlier investigation, which found these conditions to be relatively prevalent among Japanese patients with sarcoidosis [16]. Detailed definitions of each outcome are provided in Table 2, with reference to the 2024 edition of Today’s Therapeutic Guidelines and relevant prior studies [17].

**Table 2:** Codes used to define different outcomes NA: not applicable; ICD-10: International Classification of Diseases, 10th Revision

Outcome	Definition pattern	Description	
		Diagnosis	Treatment
Vertebral fracture	Diagnosis	Diagnosis codes must include the ICD-10 term for *fracture*, while the Japanese diagnostic name must include any of: *cervical*, *thoracic*, *lumbar*, *sacral*, or *vertebral*.	NA
Pneumonia	Diagnosis + Treatment	Includes the Japanese term *pneumonia*, but excludes ICD-10 codes J67x, J68x.	ATC J01x
Herpes zoster	Diagnosis	ICD-10 code or Japanese diagnostic name must include *herpes zoster* and must not include *postherpetic*.	NA
Urinary tract infection	Diagnosis + Treatment	ICD-10 code N390 or the Japanese diagnostic name includes *urinary tract infection.*	ATC J01x
Insomnia	Diagnosis + Treatment	ICD-10: F510, G470, G472, G478	Hypnotics such as ramelteon, zolpidem, suvorexant, triazolam, and brotizolam.
Hypertension	Diagnosis + Treatment	ICD-10: I10-I15	ATC C02, C03, C07, C08, C09
Hyperlipidemia	Diagnosis + Treatment	ICD-10: E78	ATC C10A-C10X
Type 2 diabetes mellitus	Diagnosis + Treatment	ICD-10: E11x-E14x	ATC A10x
Glaucoma	Diagnosis + Treatment	ICD-10: H40.0-H40.6	ATC S01E subgroups
Cataract	Diagnosis + Treatment	ICD-10: H25-H28 or the Japanese diagnostic name indicating *cataract* or *lens opacity*	ATC S01XA, or other medications based on active ingredients such as tiopronin or aldose reductase inhibitors

Statistical analyses

Matching between groups that received OCS and those that did not was performed using the MatchIt package in R (R Foundation for Statistical Computing, Vienna, Austria) with a caliper of 0.2 based on the index month, and Cox regression analysis was subsequently conducted using the survival package. The cumulative incidence of each outcome over the three years following the index month was estimated using the Kaplan-Meier method. Kaplan-Meier curves were generated based on cumulative OCS dose categories over the same three-year period: 0 mg, 1-4999 mg, and ≥5000 mg. Patients who did not experience the outcome were censored at the time of database withdrawal or at the end of the observation period. The association between cumulative OCS dose and each outcome during the three years after the index month was evaluated using multivariable Cox proportional hazards models, adjusted for age (in quartiles at the index month), sex, duration from diagnosis to the index month, and CCI. Washout periods following OCS initiation were defined as either no washout or a six-month washout. For the six-month washout period, outcomes occurring within the first five months after the index month were excluded. In the primary analysis, a six-month washout was applied for vertebral fracture and cataract, while a no-washout period was applied for all other outcomes. A six-month washout was adopted for vertebral fracture and cataract to reduce detection and reverse causation bias. Vertebral fractures are often undiagnosed and may be incidentally detected around the time of corticosteroid initiation, while corticosteroid-induced cataracts typically develop progressively over months to years. Thus, applying a washout helped ensure the temporal sequence between exposure and outcome, although this approach could underestimate early risks. To address this, we also performed a scenario analysis using a 0-month washout. That is, in a scenario analysis, these definitions were reversed, applying no washout for vertebral fracture and cataract, and a six-month washout for all other outcomes. For each outcome, patients with a history of that condition prior to the index month were excluded from the analysis. Descriptive statistics were reported as means and standard deviations (SD) or medians and interquartile ranges (IQRs) for continuous variables, and as frequencies and percentages for categorical variables. The significance level was set at two-sided 5%, and 95% confidence intervals (CIs) were reported.

All statistical analyses were conducted using R software version 4.4.0 (R Foundation for Statistical Computing, Vienna, Austria) [18]. The results of this study are reported in accordance with the REporting of studies Conducted using Observational Routinely-collected Data (RECORD) statement [19]. Only anonymized data, free of personally identifiable information, were used in this study. Therefore, in accordance with Japanese ethical guidelines [20], institutional review board approval and informed consent were not required.

## Results

**Figure 2:**
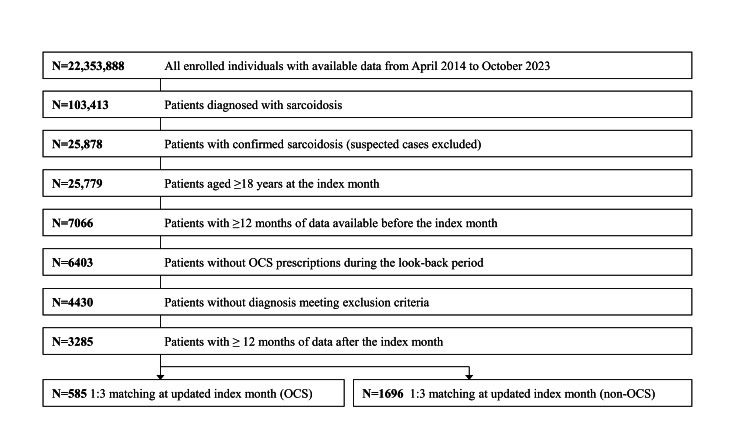
Overview of the enrolled patients Flow diagram of patient selection from a Japanese administrative claims database (April 2014-October 2023). Among all enrolled individuals, patients diagnosed with sarcoidosis were identified, and suspected cases were excluded. Additional eligibility criteria included age ≥18 years at the index month, continuous enrollment for ≥12 months before and after the index month, no prior OCS prescriptions during the 12 months prior to the index month, and no diagnosis meeting predefined exclusion criteria. Patients who received OCS were matched 1:3 with those who did not, based on the updated index month. OCS: oral corticosteroid This figure was created by the authors using data from the DeSC Healthcare claims database. No permission required.

Among 25,779 patients aged 18 years or older with sarcoidosis, 1:3 matching was performed based on the month of OCS initiation, resulting in 585 patients in the OCS group and 1696 in the non-OCS group. A total of 2281 patients were included in the analysis (Figure 2 and Table 3).

**Table 3:** Demographic and clinical characteristics by cumulative oral corticosteroid (OCS) exposure

Characteristics	Non-OCS	OCS 1-4999 mg	OCS ≥5000 mg	Overall
	(N=1696)	(N=401)	（N=184）	(N=2281)
Age				
Median (IQR)	68.0 (53.0-78.0)	67.0 (53.0-79.0)	66.0 (57.0-71.0)	68.0 (53.0-78.0)
Min-Max	20.0-94.0	18.0-94.0	20.0-89.0	18.0-94.0
Gender, n (%)				
Male	746 (44.0)	174 (43.4)	78 (42.4)	998 (43.8)
Follow-up period after the index month, month				
Median (IQR)	34.0 (22.0-50.0)	32.0 (20.0-47.0)	40.5 (27.8-54.2)	34.0 (22.0-51.0)
Min-Max	12.0-100.0	12.0-91.0	12.0-91.0	12.0-100.0
Number of event during look-back period, n (%)				
Vertebral fracture	59 (3.5)	13 (3.2)	5 (2.7)	77 (3.4)
Pneumonia	78 (4.6)	27 (6.7)	9 (4.9)	114 (5.0)
Herpes zoster	52 (3.1)	18 (4.5)	6 (3.3)	76 (3.3)
Urinary tract infection	28 (1.7)	6 (1.5)	2 (1.1)	36 (1.6)
Insomnia	265 (15.6)	66 (16.5)	34 (18.5)	365 (16.0)
Hypertension	789 (46.5)	202 (50.4)	95 (51.6)	1086 (47.6)
Hyperlipidemia	545 (32.1)	127 (31.7)	68 (37.0)	740 (32.4)
Diabetes mellitus	586 (34.6)	132 (32.9)	76 (41.3)	794 (34.8)
Glaucoma	306 (18.0)	75 (18.7)	31 (16.8)	412 (18.1)
Cataract	498 (29.4)	129 (32.2)	62 (33.7)	689 (30.2)
Cumulative OCS dose after the index month, mg				
1 year				
Median (IQR)	0.0 (0.0-0.0)	1245.0 (220.0-2625.0)	4810.5 (4067.5-5938.8)	0.0 (0.0-14.0)
2 year				
Median (IQR)	0.0 (0.0-0.0)	1441.0 (255.0-3017.5)	6357.0 (5403.0-8222.5)	0.0 (0.0-14.0)
3 year				
Median (IQR)	0.0 (0.0-0.0)	1495.0 (255.0-3122.0)	7693.8 (6074.1-9346.1)	0.0 (0.0-14.0)
Distribution of cumulative OCS dose after the index month, n (%)				
1 year				
0 mg	1696 (100%)	0 (0)	0 (0)	1696 (74.4)
1-4999 mg	0 (0)	401 (100)	104 (56.5)	505 (22.1)
≥5000 mg	0 (0)	0 (0)	80 (43.5)	80 (3.5)
2 year				
0 mg	1696 (100)	0 (0)	0 (0)	1696 (74.4)
1-4999 mg	0 (0)	401 (100)	23 (12.5)	424 (18.6)
≥5000 mg	0 (0)	0 (0)	161 (87.5)	161 (7.1)
3 year				
0 mg	1696 (100)	0 (0)	0 (0)	1696 (74.4)
1-4999 mg	0 (0)	401 (100)	0 (0)	401 (17.6)
≥5000 mg	0 (0)	0 (0)	184 (100)	184 (8.1)

Patient characteristics are summarized in Table 3. The median age in the overall cohort was 68 years (IQR: 53.0-78.0), 67.0 years in the OCS 1-4999 mg group, and 66.0 years in the ≥5000 mg group. The number of male patients was 998 (43.8%) in the overall cohort, 174 (43.4%) in the 1-4999 mg group, and 78 (42.4%) in the ≥5000 mg group. The three-year cumulative OCS dose was 1495 mg in the 1-4999 mg group and 7694 mg in the ≥5000 mg group.

**Figure 3:**
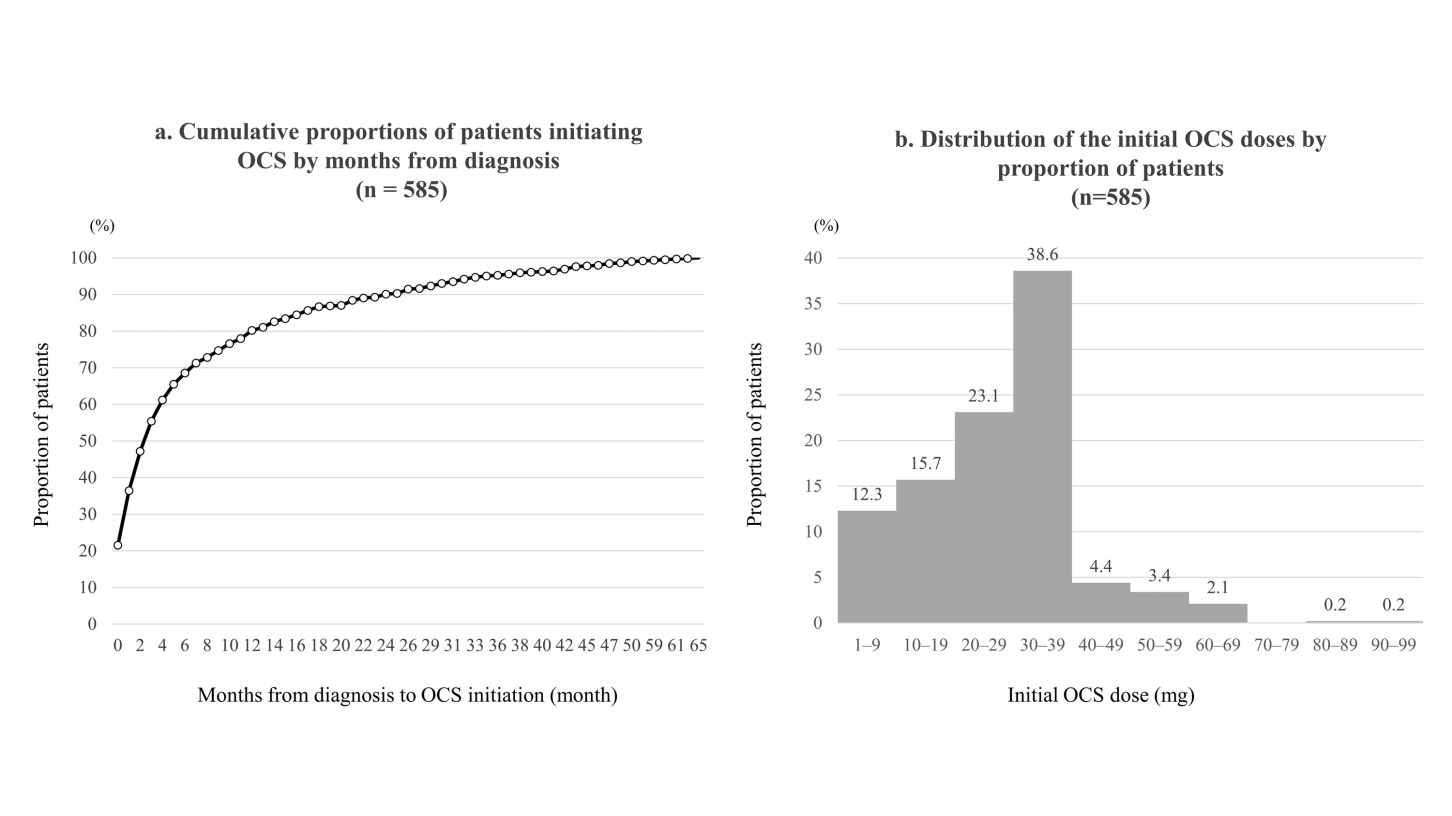
Distribution of patients by time to oral corticosteroid (OCS) initiation and initial dose (a) Cumulative proportions of patients initiating OCS therapy according to the number of months from diagnosis. A total of 401 (68.5%) patients initiated OCS within six months of diagnosis. (b) Distribution of initial OCS doses: 1-9 mg in 72 (12.3%) patients, 10-19 mg in 92 (15.7%), 20-29 mg in 135 (23.1%), 30-39 mg in 226 (38.6%), 40-49 mg in 26 (4.4%), 50-59 mg in 20 (3.4%), 60-69 mg in 12 (2.1%), 80-89 mg in 1 (0.2%), and 90-99 mg in 1 (0.2%). Both analyses were conducted among patients who initiated OCS (n = 585). This figure was created by the authors using data from the DeSC Healthcare claims database. No permission required.

The distribution of patients by time to OCS initiation and initial dose among those prescribed OCS is shown in Figure 3. Among patients who initiated OCS within the three-year follow-up period, 126 (21.5%) initiated treatment in the same month as their sarcoidosis diagnosis, and 401 (68.5%) initiated treatment within six months after diagnosis. The most frequently prescribed initial OCS dose was 30 mg, accounting for 220 (37.6%) of patients who received OCS.

**Figure 4:**
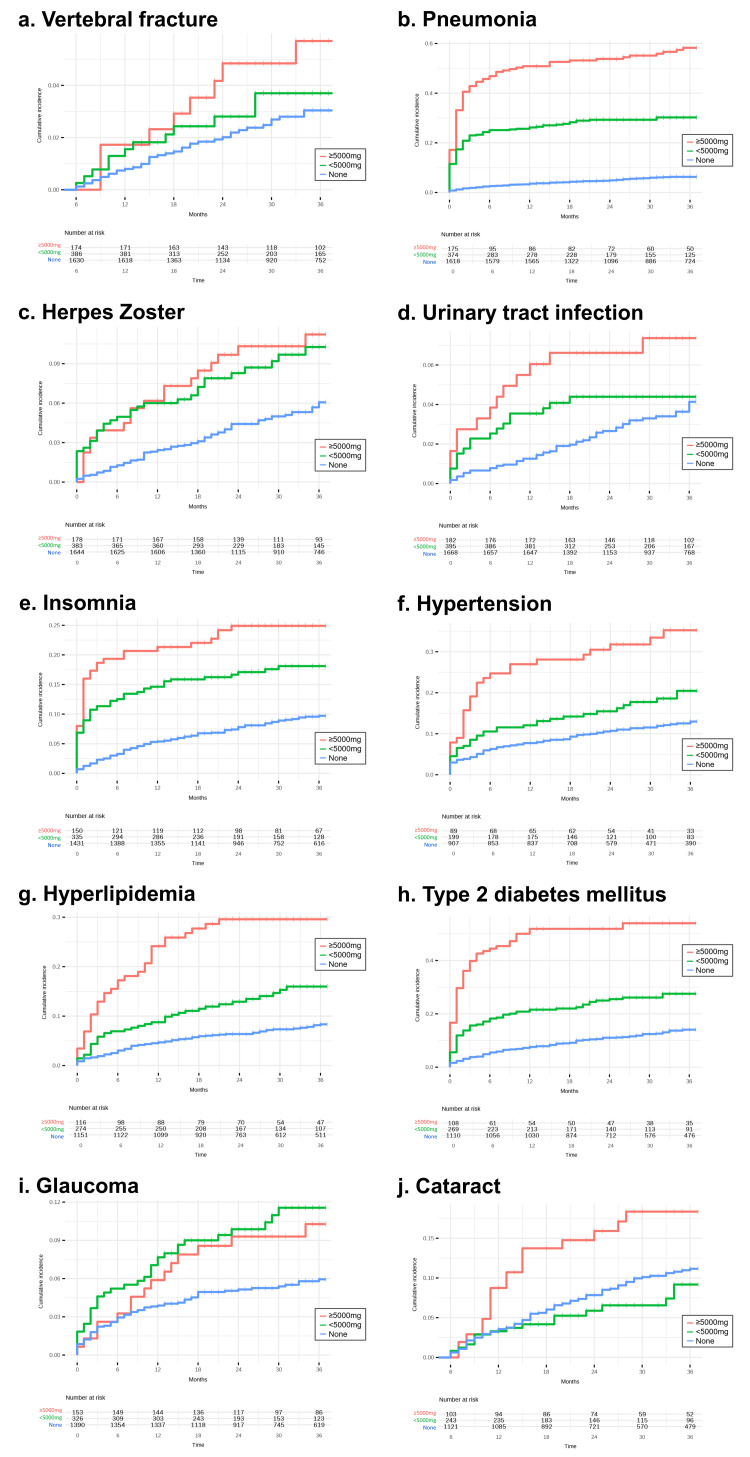
Kaplan-Meier curves of the cumulative incidence of different outcomes by cumulative oral corticosteroid (OCS) exposure Kaplan-Meier curves showing the cumulative incidence of each outcome stratified by three-year cumulative OCS dose categories: none, 1-4999 mg, and ≥5000 mg (prednisone-equivalent). Outcomes include (a) vertebral fracture, (b) pneumonia, (c) herpes zoster, (d) urinary tract infection, (e) insomnia, (f) hypertension, (g) hyperlipidemia, (h) type 2 diabetes mellitus, (i) glaucoma, and (j) cataract. Numbers at risk are shown below each panel. This figure was created by the authors using data from the DeSC Healthcare claims database. No permission required.

Kaplan-Meier curves of cumulative incidence are shown in Figure 4.

The cumulative incidence of each outcome is shown in Table 4.

**Table 4:** Cumulative incidence of each outcome according to the cumulative oral corticosteroid (OCS) dose Data are shown as n (%). Cumulative incidence at <12, <24, and <36 months from the index month, stratified by three-year cumulative OCS dose categories: none, 1-4999 mg, and ≥5000 mg (prednisone-equivalent). For vertebral fracture and cataract, a six-month washout period was applied. In these outcomes, the denominator was the number of patients remaining at six months after the index month (including the index month), and events occurring within the first five months were excluded. For all other outcomes, no washout period was applied. In these outcomes, the denominator was all eligible patients at the index month. Numerators are the number of events observed during each observation window. Patients with a prior history of the respective outcome before the index month were excluded from that outcome’s analysis.

Outcome	Cumulative OCS dose over three years	n	<12 months		<24 months		<36 months	
Vertebral fracture	None	1630	12	0.7%	29	1.8%	40	2.5%
	1-4999 mg	386	5	1.3%	10	2.6%	12	3.1%
	≥5000 mg	174	3	1.7%	7	4.0%	9	5.2%
	Total	2190	20	0.9%	46	2.1%	61	2.8%
Pneumonia	None	1618	53	3.3%	74	4.6%	90	5.6%
	1-4999 mg	374	96	25.7%	108	28.9%	110	29.4%
	≥5000 mg	175	89	50.9%	94	53.7%	100	57.1%
	Total	2167	238	11.0%	276	12.7%	300	13.8%
Herpes zoster	None	1644	38	2.3%	67	4.1%	79	4.8%
	1-4999 mg	383	23	6.0%	30	7.8%	34	8.9%
	≥5000 mg	178	11	6.2%	17	9.6%	19	10.7%
	Total	2205	72	3.3%	114	5.2%	132	6.0%
Urinary tract infection	None	1668	21	1.3%	41	2.5%	51	3.1%
	1-4999 mg	395	14	3.5%	17	4.3%	17	4.3%
	≥5000 mg	182	10	5.5%	12	6.6%	13	7.1%
	Total	2245	45	2.0%	70	3.1%	81	3.6%
Insomnia	None	1431	76	5.3%	102	7.1%	121	8.5%
	1-4999 mg	335	49	14.6%	55	16.4%	58	17.3%
	≥5000 mg	150	31	20.7%	37	24.7%	37	24.7%
	Total	1916	156	8.1%	194	10.1%	216	11.3%
Hypertension	None	907	70	7.7%	92	10.1%	103	11.4%
	1-4999 mg	199	24	12.1%	30	15.1%	36	18.1%
	≥5000 mg	89	24	27.0%	27	30.3%	30	33.7%
	Total	1195	118	9.9%	149	12.5%	169	14.1%
Hyperlipidemia	None	1151	52	4.5%	71	6.2%	83	7.2%
	1-4999 mg	274	24	8.8%	34	12.4%	39	14.2%
	≥5000 mg	116	28	24.1%	34	29.3%	34	29.3%
	Total	1541	104	6.7%	139	9.0%	156	10.1%
Type 2 diabetes mellitus	None	1110	80	7.2%	117	10.5%	137	12.3%
	1-4999 mg	269	56	20.8%	65	24.2%	69	25.7%
	≥5000 mg	108	54	50.0%	56	51.9%	58	53.7%
	Total	1487	190	12.8%	238	16.0%	264	17.8%
Glaucoma	None	1390	53	3.8%	68	4.9%	74	5.3%
	1-4999 mg	326	23	7.1%	31	9.5%	34	10.4%
	≥5000 mg	153	9	5.9%	14	9.2%	15	9.8%
	Total	1869	85	4.5%	113	6.0%	123	6.6%
Cataract	None	1121	36	3.2%	81	7.2%	102	9.1%
	1-4999 mg	243	8	3.3%	13	5.3%	17	7.0%
	≥5000 mg	103	9	8.7%	15	14.6%	18	17.5%
	Total	1467	53	3.6%	109	7.4%	137	9.3%

The results of the multivariable Cox proportional hazards analysis assessing the association between cumulative OCS dose over a three-year period and each outcome are presented in Table 5. For vertebral fracture, the hazard ratio (HR) was 1.43 (95% CI: 0.74-2.77) in the 1-4999 mg group and 2.13 (95% CI: 1.01-4.48) in the ≥5000 mg group, compared with the non-OCS group. For pneumonia, the HR was 5.87 (95% CI: 4.43-7.79) in the 1-4999 mg group and 13.47 (95% CI: 10.01-18.12) in the ≥5000 mg group. Herpes zoster, insomnia, hypertension, hyperlipidemia, and type 2 diabetes mellitus were all significantly associated with both 1-4999 mg and ≥5000 mg cumulative OCS exposure. Glaucoma showed a significant association only in the 1-4999 mg group, while cataract was not significantly associated in either group. Results from the scenario analysis using alternative washout period definitions showed similar trends and are also presented in Table 5. A visual overview of the study design and key findings is provided in Appendix A.

**Table 5:** Adjusted hazard ratios for steroid-related outcomes by cumulative oral corticosteroid (OCS) dose over a three-year period Hazard ratios (HRs) were calculated using the Cox proportional hazards models adjusted for age (quartiles at the index month), sex, duration from diagnosis to the index month, and Charlson Comorbidity Index. Washout periods following OCS initiation were defined as either no-washout or a washout period of six months. For the six-month washout period, events occurring within the first five months after the index month were excluded. In the primary analysis, vertebral fracture and cataract were assigned a six-month washout period, while no washout period was applied for all other outcomes. As a scenario analysis, we reversed these definitions: applying a no-washout period for vertebral fracture and cataract, and a six-month washout for all other outcomes. For each outcome, patients who had experienced the corresponding event prior to the index month were excluded from the analysis. HRs with P < 0.05 are marked with an asterisk (*). HR: hazard ratio; CI: confidence interval; LCL: lower confidence limit; UCL: upper confidence limit

Outcome	Variable (three-year cumulative OCS dose)	Reference	Primary analysis			Scenario analysis		
			HR	95% CI		HR	95% CI	
				LCL	UCL		LCL	UCL
Vertebral fracture	1-4999 mg	Non-OCS	1.43	0.74	2.77	1.39	0.76	2.55
	≥5000 mg		2.13*	1.01	4.48	2.87*	1.54	5.32
Pneumonia	1-4999 mg	Non-OCS	5.87*	4.43	7.79	1.95*	1.14	3.32
	≥5000 mg		13.47*	10.01	18.12	5.75*	3.39	9.73
Herpes zoster	1-4999 mg	Non-OCS	1.85*	1.24	2.78	1.26	0.65	2.46
	≥5000 mg		1.81*	1.09	3.01	1.35	0.63	2.91
Urinary tract infection	1-4999 mg	Non-OCS	1.20	0.69	2.09	0.72	0.34	1.55
	≥5000 mg		1.98*	1.06	3.69	1.34	0.59	3.03
Insomnia	1-4999 mg	Non-OCS	2.09*	1.52	2.87	1.00	0.59	1.70
	≥5000 mg		2.83*	1.93	4.16	0.93	0.44	1.95
Hypertension	1-4999 mg	Non-OCS	1.72*	1.17	2.53	1.48	0.82	2.67
	≥5000 mg		2.86*	1.86	4.37	1.84	0.88	3.84
Hyperlipidemia	1-4999 mg	Non-OCS	2.08*	1.41	3.06	1.66	0.99	2.80
	≥5000 mg		3.76*	2.49	5.66	2.86*	1.62	5.07
Type 2 diabetes mellitus	1-4999 mg	Non-OCS	2.32*	1.73	3.12	1.28	0.80	2.04
	≥5000 mg		5.56*	4.07	7.61	1.93*	1.02	3.63
Glaucoma	1-4999 mg	Non-OCS	1.92*	1.28	2.90	1.89*	1.06	3.37
	≥5000 mg		1.41	0.80	2.48	2.13*	1.07	4.24
Cataract	1-4999 mg	Non-OCS	0.81	0.48	1.36	1.20	0.86	1.67
	≥5000 mg		1.46	0.87	2.43	1.68*	1.17	2.41

## Discussion

In this study, we investigated the cumulative OCS dose in newly diagnosed patients with sarcoidosis and examined its association with OCS-related complications over a three-year period. Approximately 70% of patients prescribed OCS initiated treatment within six months of diagnosis, with an initial daily dose of 30 mg prescribed to approximately 38% of patients. The median three-year cumulative OCS doses were 1495 mg (1-4999 mg group) and 7694 mg (≥5000 mg group). Notably, a higher cumulative OCS dose was significantly associated with an increased risk of steroid-related complications compared with no OCS exposure. To our knowledge, this is the first large-scale study to describe real-world OCS prescribing patterns and related complications in Japanese patients with sarcoidosis using a national claims database.

In Japan, there is currently no standardized protocol for initial OCS dosing in sarcoidosis. The Japanese guidelines [21] recommend an initial prednisone dose of 20-30 mg/day, advocating for the lowest effective dose. Our findings suggest that current prescribing practices align closely with these guidelines. A South Korean study reported an average initial prednisone-equivalent dose of 33.0 ± 25.7 mg/day, aligning with our results [12]. To date, this is the first study to document initial steroid prescribing patterns for sarcoidosis in Japan. In contrast, a US study highlighted wide variability in prednisone dosing, attributed to clinical heterogeneity [11]. In this study, the median three-year cumulative OCS dose was 2990 mg (IQR: 707.5-5716.5 mg), indicating substantial variations in prescribing that may reflect differences in disease severity or patient characteristics. However, the claims database used does not include direct indicators of disease severity, limiting assessment of dosing appropriateness. Future studies incorporating clinical data are needed.

In this study, we observed a significantly increased vertebral fracture risk in patients with a three-year cumulative OCS dose of ≥5000 mg, while no increased risk was observed at doses of 1-4999 mg. A previous study in giant cell arteritis [14] reported a 4% increase in fracture risk (HR = 1.04) per additional 1000 mg cumulative prednisone-equivalent dose, suggesting a dose-dependent effect of corticosteroids on bone fragility. Our findings similarly suggest a dose-dependent association between corticosteroid exposure and fracture risk, highlighting the importance of implementing bone-preventive strategies and considering steroid-sparing alternatives. However, fracture risk is influenced by multiple factors beyond corticosteroid use, such as bone mineral density, nutritional status, physical activity, and systemic inflammation, which were not captured in this study. Future research incorporating clinical and lifestyle data is needed for a more comprehensive risk evaluation.

Regarding pneumonia, patients who received 1-4999 mg or ≥5000 mg of OCS had a significantly higher pneumonia risk than non-users. These findings are consistent with those of a Swedish registry-based study [10], which reported that patients with sarcoidosis had a significantly elevated risk of serious infections compared with the general population (adjusted HR (aHR): 1.81), with an approximately threefold higher risk (aHR: 3.04) among those treated with immunosuppressive therapy, including corticosteroids, around the time of diagnosis. While the Swedish study focused on the presence or absence of immunosuppressive therapy, our study emphasizes a dose-dependent association between cumulative OCS exposure and infection risk, underscoring the need for vigilant corticosteroid risk management. Furthermore, the Swedish study found the infection risk was highest during the first two years post-diagnosis. Similarly, pneumonia cases in our study were concentrated in the early phase following OCS initiation, emphasizing the need for robust infection prevention strategies during initial treatment. Prior research has shown that lower baseline forced vital capacity (FVC) is associated with a higher risk of pneumonia-related hospitalization in sarcoidosis, with a 10% predicted FVC decrease corresponding to an HR of 1.15 [22]. As corticosteroids are more frequently prescribed in patients with higher disease activity, fully accounting for confounding by disease severity is challenging, and our findings necessitate cautious interpretation. Moreover, herpes zoster and urinary tract infections also showed elevated risks with increasing cumulative OCS exposure. Herpes zoster occurred significantly more frequently in both dose groups, while urinary tract infection was significantly associated with a cumulative dose of ≥5000 mg. These results further support the need for close monitoring and proactive infection control in patients receiving prolonged corticosteroid therapy.

An increased risk of type 2 diabetes mellitus and insomnia was observed in patients with cumulative OCS doses of 1-4999 mg and ≥5000 mg compared with non-users. A UK study on inflammatory diseases, including rheumatoid arthritis, reported a dose-dependent increase in diabetes risk associated with corticosteroid use [5], emphasizing the need for regular follow-up to enable early diabetes detection and management, irrespective of dose or duration. Similarly, a US survey of patients using OCS for over 60 days found that insomnia risk rose with cumulative prednisone dose [23]. Notably, even among those averaging <7.5 mg/day, each 1 mg increase was significantly associated with higher odds of insomnia (OR = 1.14), indicating a heightened sensitivity to daily glucocorticoid exposure. In our study, insomnia risk was significantly associated with cumulative OCS dose in the primary analysis, including at doses of 1-4999 mg over three years. However, as most events occurred within six months of OCS initiation, no significant difference was observed in the six-month washout analysis. These findings highlight the need for careful corticosteroid management to mitigate early complications such as insomnia.

An increased risk of glaucoma was observed in patients with a cumulative OCS dose of 1-4999 mg (HR = 1.92); however, not in those receiving ≥5000 mg (HR = 1.41). This may be partly attributable to the limited number of glaucoma events in the ≥5000 mg group, potentially reducing the statistical power to detect a significant association. Cataract - a well-known delayed complication of long-term corticosteroid therapy - occurred within six months of OCS initiation in patients receiving ≥5000 mg. This may reflect accelerated cataract development due to high-dose corticosteroid exposure or earlier detection due to regular ophthalmologic monitoring; however, these interpretations remain speculative. A previous study [14] reported a 5% increase in glaucoma risk and a 3% increase in cataract risk per 1000 mg increase in cumulative prednisone-equivalent dose. Our study did not identify significant trends; however, the findings suggest that both complications may adversely impact older adults' QOL, emphasizing the importance of regular ophthalmologic monitoring during corticosteroid therapy.

Limitations

This study has some limitations. First, diagnoses and outcome events were identified using administrative claims data, which may not always correspond to clinical diagnoses. However, to improve the validity of outcome identification, we required not only diagnostic codes but also the concurrent prescription of relevant therapeutic agents. Second, we were unable to assess patients’ actual adherence to prescribed OCS regimens. Therefore, differences between prescribed and actual medication use may have influenced the outcomes. Third, the potential influence of unmeasured confounding factors, such as disease severity, smoking, alcohol consumption, and dietary habits, cannot be excluded. These lifestyle and clinical variables were not available in the claims database and may have affected the outcomes. Finally, the observation period was limited to three years from the index month, precluding evaluation of long-term effects beyond this timeframe. The evaluation period of the present study overlapped in part with the COVID-19 pandemic. Because sarcoidosis is a designated intractable disease, the frequency of hospital visits and the treatment regimens were largely unchanged. However, some outcomes may have been underestimated due to patients refraining from visiting hospitals.

## Conclusions

To the best of our knowledge, this study is the first to characterize real-world OCS use in sarcoidosis and to demonstrate, using a large-scale Japanese claims database, that OCS use is associated with an increased risk of related complications. Our findings suggest that adverse events, including fractures, infections, glucose metabolism disorders, and insomnia, occur in a dose-dependent manner, underscoring the need for treatment strategies and preventive measures tailored to individual risk. Our previous research has also reported limited use of non-steroidal immunosuppressants in Japan. These findings highlight the importance of expanding treatment options beyond corticosteroids, including immunosuppressants and biologic agents. Further studies are needed to determine whether such alternatives can reduce the risk of OCS-related complications.

## Figures and Tables

**Figure 5:**
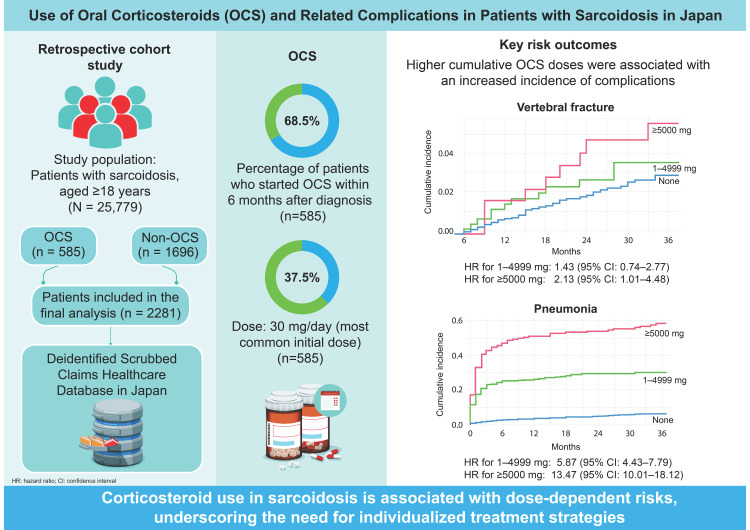
Graphical abstract This graphical abstract was created by Editage (Cactus Communications) and is used under license for this article only. Redistribution, resale, sublicensing, or use of individual design elements in other projects is prohibited without prior permission from the copyright holder © Editage.

## Appendices

Appendix A 

## References

[1] Valeyre D, Prasse A, Nunes H, Uzunhan Y, Brillet PY, Müller-Quernheim J (2014). Sarcoidosis. Lancet.

[2] Sève P, Pacheco Y, Durupt F (2021). Sarcoidosis: a clinical overview from symptoms to diagnosis. Cells.

[3] Baughman RP, Valeyre D, Korsten P (2021). ERS clinical practice guidelines on treatment of sarcoidosis. Eur Respir J.

[4] Dalal AA, Duh MS, Gozalo L (2016). Dose-response relationship between long-term systemic corticosteroid use and related complications in patients with severe asthma. J Manag Care Spec Pharm.

[5] Wu J, Mackie SL, Pujades-Rodriguez M (2020). Glucocorticoid dose-dependent risk of type 2 diabetes in six immune-mediated inflammatory diseases: a population-based cohort analysis. BMJ Open Diabetes Res Care.

[6] Iki M, Fujimori K, Nakatoh S (2024). Average daily glucocorticoid dose, number of prescription days, and cumulative dose in the initial 90 days of glucocorticoid therapy are associated with subsequent hip and clinical vertebral fracture risk: a retrospective cohort study using a nationwide health insurance claims database in Japan. Osteoporos Int.

[7] Chaudhary NS, Donnelly JP, Moore JX, Baddley JW, Safford MM, Wang HE (2017). Association of baseline steroid use with long-term rates of infection and sepsis in the REGARDS cohort. Crit Care.

[8] Van Staa TP, Leufkens HG, Abenhaim L, Zhang B, Cooper C (2000). Use of oral corticosteroids and risk of fractures. J Bone Miner Res.

[9] Balasubramanian A, Wade SW, Adler RA, Saag K, Pannacciulli N, Curtis JR (2018). Glucocorticoid exposure and fracture risk in a cohort of US patients with selected conditions. J Bone Miner Res.

[10] Rossides M, Kullberg S, Eklund A, Di Giuseppe D, Grunewald J, Askling J, Arkema EV (2020). Risk of first and recurrent serious infection in sarcoidosis: a Swedish register-based cohort study. Eur Respir J.

[11] Simmering J, Stapleton EM, Polgreen PM, Kuntz J, Gerke AK (2021). Patterns of medication use and imaging following initial diagnosis of sarcoidosis. Respir Med.

[12] Yoon HY, Kim HM, Kim YJ, Song JW (2018). Prevalence and incidence of sarcoidosis in Korea: a nationwide population-based study. Respir Res.

[13] Quan H, Li B, Couris CM (2011). Updating and validating the Charlson comorbidity index and score for risk adjustment in hospital discharge abstracts using data from 6 countries. Am J Epidemiol.

[14] Broder MS, Sarsour K, Chang E, Collinson N, Tuckwell K, Napalkov P, Klearman M (2016). Corticosteroid-related adverse events in patients with giant cell arteritis: a claims-based analysis. Semin Arthritis Rheum.

[15] Sullivan PW, Ghushchyan VH, Globe G, Schatz M (2018). Oral corticosteroid exposure and adverse effects in asthmatic patients. J Allergy Clin Immunol.

[16] Miyashita K, Hashimoto K, Maeda S, Suda T (2025). Current epidemiological and clinical features of sarcoidosis in Japan: a nationwide claims data study. Cureus.

[17] Jansson C, Alexanderson K, Kecklund G, Akerstedt T (2013). Clinically diagnosed insomnia and risk of all-cause and diagnosis-specific disability pension: a nationwide cohort study. Sleep Disord.

[18] (2025). The R Project for statistical computing. https://www.R-project.org/.

[19] Benchimol EI, Smeeth L, Guttmann A (2015). The REporting of studies Conducted using Observational Routinely-collected health Data (RECORD) statement. PLoS Med.

[20] (2025). Ethical guidelines for medical and health research involving human subjects. https://warp.da.ndl.go.jp/info:ndljp/pid/11656272/www.mhlw.go.jp/file/06-Seisakujouhou-10600000-Daijinkanboukouseikagakuka/0000080278.pdf.

[21] Japanese Society of Sarcoidosis/Granulomatous Disease (2023). Sarcoidosis Treatment Guide 2023 [Book in Japanese]. Practical Guide for the Diagnosis and Treatment of Sarcoidosis 2023 (In Japanese).

[22] Ungprasert P, Crowson CS, Matteson EL (2017). Sarcoidosis increases risk of hospitalized infection. A population-based study, 1976-2013. Ann Am Thorac Soc.

[23] Curtis JR, Westfall AO, Allison J (2006). Population-based assessment of adverse events associated with long-term glucocorticoid use. Arthritis Rheum.

